# miR-151a enhances Slug dependent angiogenesis

**DOI:** 10.18632/oncotarget.27331

**Published:** 2020-06-09

**Authors:** Douglas Jury, Iben Daugaard, Katie J. Sanders, Lise Lotte Hansen, Dritan Agalliu, Irene Munk Pedersen

**Affiliations:** ^1^Department of Molecular Biology and Biochemistry, School of Biological Sciences, University of California, Irvine, CA 92697, USA; ^2^Department of Biomedicine, Aarhus University, Aarhus DK-8000, Denmark; ^3^Department of Pathology, Aarhus University Hospital, Aarhus DK-8200, Denmark; ^4^Department of Neurology, Columbia University Irving Medical Center, New York, NY 10032, USA; ^5^Department of Pathology and Cell Biology, Columbia University Irving Medical Center, New York, NY 10032, USA; ^6^Scintillon Institute, San Diego, CA 92121, USA

**Keywords:** angiogenesis, miR-151a, Slug, tumor microenvironment, endothelial cell

## Abstract

MicroRNAs (miRs) are small non-coding RNAs, that modulate cognate gene expression either by inducing mRNA degradation or by blocking translation, and play crucial and complex roles in tissue homeostasis and during disease initiation and progression. The sprouting of new blood vessels by angiogenesis is critical in vascular development and homeostasis and aberrant angiogenesis is associated with pathological conditions such as ischemia and cancer. We have previously established that miR-151a functions as an onco-miR in non-small cell lung cancer (NSCLC) cells by inducing partial EMT and enhancing tumor growth. Here, we identify anti-miR-151a as a molecule that promotes endothelial cell contacts and barrier properties, suggesting that miR-151a regulates cell-cell junctions. We find that induced miR-151a expression enhances endothelial cell motility and angiogenesis and these functions depend on miR-151a-induced Slug levels. Moreover, we show that miR-151a overexpression enhances tumor-associated angiogenesis in 3D vascularized tumor spheroid assays. Finally, we verify that miR-151a is expressed in the vasculature of normal lung and NSCLC tissue. Our results suggest that miR-151a plays multi-faceted roles in the lung, by regulating multiple functions (cell growth, motility, partial EMT and angiogenesis) in distinct cell types.

## INTRODUCTION

Angiogenesis, or the growth of new networks of blood vessels from existing vessels, is an important natural process used for growth, healing and reproduction. Blood vessels form one of the body’s largest surfaces, and they serve as a critical regulatory interface between the circulating components (nutrients, immune cells, antibodies or toxins) and various organ microenvironments [1–4]. Proper regulation of angiogenesis is essential not only for developing an adult’s healthy organs to support growth and metabolism, but also for disease progression since abnormal vessel growth and/or function are hallmarks of cancer, ischemic and chronic inflammatory diseases [1, 5].

Snail, Slug, TWIST1, ZEB1 and ZEB2 are considered as core epithelial to mesenchymal transition (EMT) transcription factors (EMT-TFs). EMT-TFs are evolutionarily conserved and they are required and essential both during embryonic development and in the adult organism especially during wound healing [6–9]. Individual EMT-TFs regulate the expression of common and unique target genes, which they can either repress or activate [6, 8, 10, 11]. In addition to regulating EMT, this family of transcription factors can also activate and maintain stemness traits, prevent DNA damage and irradiation-induced cell death, thereby promoting radioresistance [7, 10, 12–14]. Depletion of Snail results in an embryonic lethal phenotype caused by early defective gastrulation, while Slug knock-out mice are viable [15, 16]. Slug and Snail may regulate a distinct but overlapping set of genes and therefore Snail may compensate for Slug in the Slug-knock-out context.

Small non-coding RNAs (~22nt) known as microRNA (miRs) have been established as significant regulators of our transcriptome by targeting messenger RNA (mRNA) for degradation or through translational repression [17, 18]. miRs can function as master regulators of networks and as such are suitable for orchestrating the complex and dynamic process of vascular development. In contrast, dysregulation of miRs can be exploited during pathological angiogenesis including cancer, inflammation and cardiovascular diseases and contribute to disease progression [19–24]. There is a need to characterize the repertoire of master miR regulators in normal and diseased microenvironments to further understand how to restore the delicate balance of cell homeostasis when it has been lost.

In this study, we report the results from a novel anti-miR library screen strategy utilized to identify miRs that regulate endothelial cell contacts and barrier properties. We find that anti-miR-375 and anti-miR-151a strengthen cell-cell contact and endothelial cell barrier in primary lung endothelial cells, relative to control samples. We determine that induced miR-151a expression enhances endothelial cell angiogenesis and this process depends on increased Slug protein levels. Taken together, our findings suggest that miR-151a plays multifaceted roles in different cells types of the human lung, thereby demonstrating the complex regulatory potentials of these small molecules.

## RESULTS

### Identification of miRs regulating endothelial cell contacts

**Figure 1:**
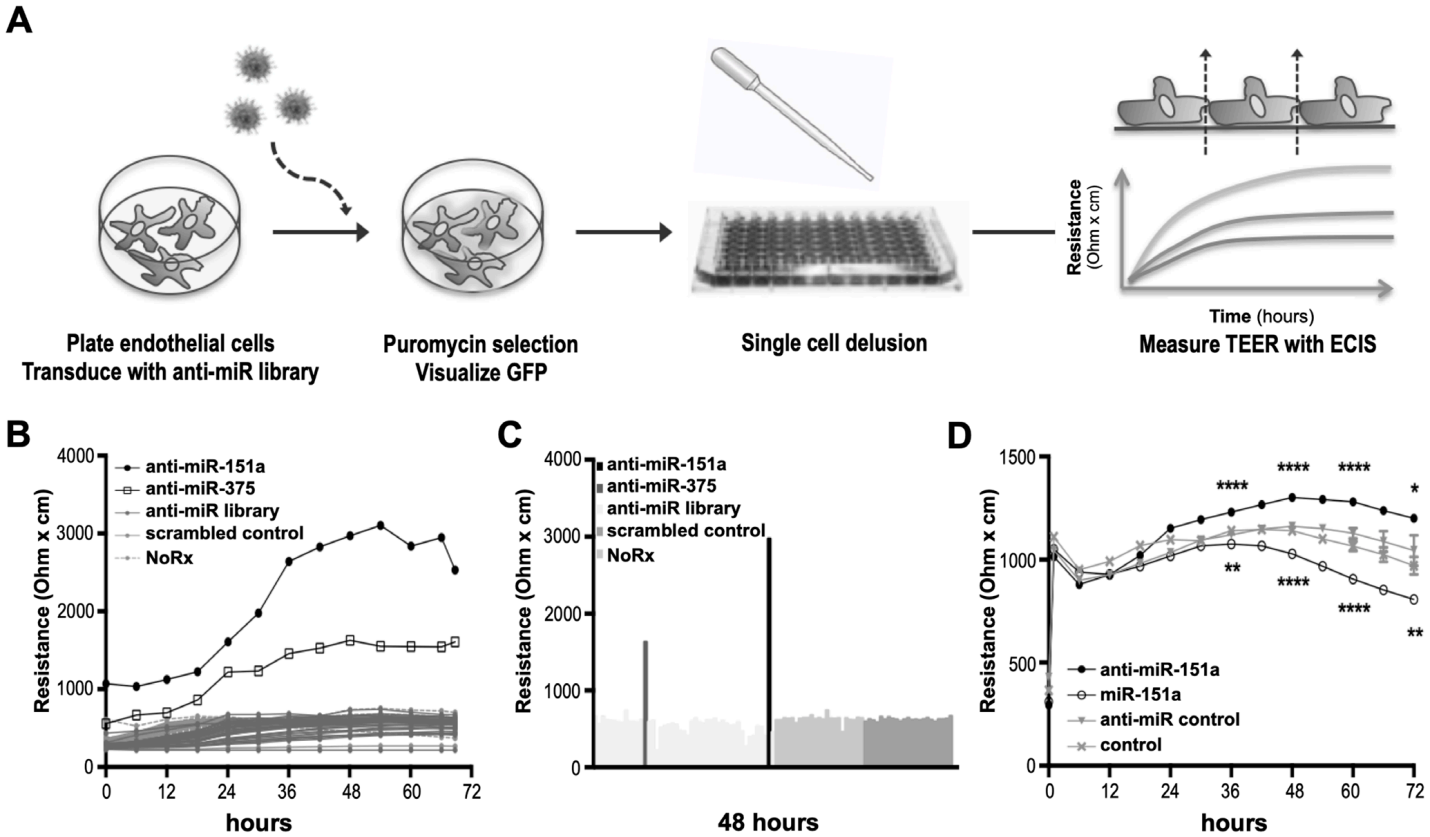
Identification of miRs regulating cell-cell contact. (**A**) Schematic representation of the anti-miR library barrier strength screen in lung endothelial cells (LECs). (**B**) Mouse LECs were transduced with a lentiviral miR-neutralizing shRNA library, selected, and clonally expanded. Transendothelial electrical resistance (TEER) was measured across LECs using the ECIS system for 72 hr. (**C**) Representation of TEER measurements after 48 hr. (**D**) Human LECs were transduced with high titer anti-miR-151a, miR-151a or control miRs and TEER was measured for 72 hrs. (*n* = 1 biological replicate, 8 technical replicates, ^*^ *p* < 0.05, ^**^ *p* < 0.01, ^****^ *p* < 0.0001). Shown as mean ± SEM. Statistical significance was assessed using unpaired student’s *t*-test.

Since miRs function in complex networks to regulate gene expression both at the mRNA and protein levels, it could be predicted that miRs regulating cell-cell contact and endothelial cell properties would also affect angiogenesis and thus be involved in the regulation of vascular development, wound healing and cancer angiogenesis and metastasis [1, 5, 25]. Proteins that form adherens and tight junctions between endothelial cells regulate paracellular barrier permeability that can be measured by trans-endothelial electrical resistance (TEER) across an adherent cell monolayer (Figure 1A) [26]. To identify miRs with novel roles in endothelial cell contact, we designed a lentiviral-based knockdown screen strategy in which libraries of expressed short hairpin RNAs (shRNAs) with anti-miR activity were used to neutralize endogenously expressed miRs in primary mouse lung endothelial cells (mLECs). This approach favors a physiologically relevant response by avoiding potential artifacts resulting from ectopic overexpression occurring in miR-mimic library screens. Non-treated mLECs and cells transduced with scrambled anti-control lentivirus were included as controls. Transduced and selected cells were diluted to single cells in 96-well plates (Figure 1A). When reaching confluence, TEER was measured over 72 hours using the electric cell-substrate impedance sensing (ECIS) system (Figure 1B). By cloning and sequencing we identified two anti-miRs, which significantly increase TEER across the endothelial cell (EC) monolayer, namely anti-miR-151a and anti-miR-375 (Figure 1B, 1C). We decided to focus our effort on miR-151a, since the oncogenic potential of miR-375 has already been extensively studied in cancers, including NSCLC [27, 28]. We validated our primary screen data by generating miR-151a, anti-miR-151a and control shRNA lentiviruses at high titer, transducing them into human LECs, and then selecting with puromycin. We observed a significant increase in TEER in cells expressing anti-miR-151a as compared to control miR expressing cells, as expected (Figure 1D). In contrast, hLECs over-expressing miR-151a significantly decreased barrier properties as determined by TEER measurements, relative to miR controls (Figure 1D). These combined results show that miR-151a plays an important role in the regulation of barrier properties of lung endothelial cells.

### The levels of miR-151a in HUVECs balance endothelial cell barrier versus migration and invasion properties

**Figure 2:**
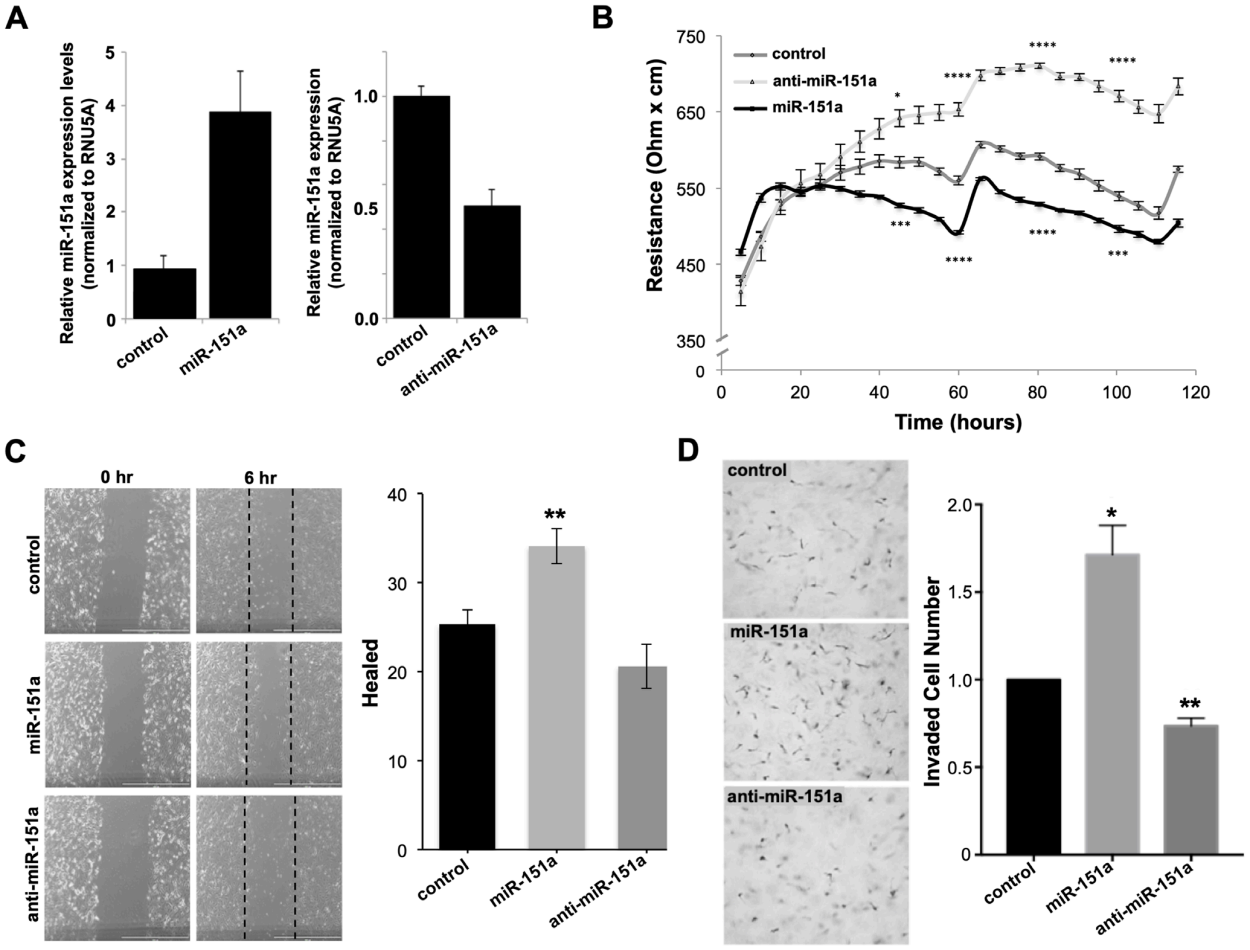
miR-151a decreases TEER and increases the motility potential of HUVEC cells. (**A**) The relative expression of miR-151a was determined by RT-qPCR in HUVEC stably expressing miR control (control), miR-151a or anti-miR-151a, relative to RNU5A expression (**B**) Stably miR-modulated HUVECs (miR control, miR-151a or anti-miR-151a) were plated in 96-well plates and transendothelial electrical resistance (TEER) was measured across the endothelial cell monolayer using the ECIS system for 72 hr. (**C**) Stably miR-modulated HUVECs were pre-treated with mitomycin c for 2 hrs, then cell migration was analyzed by scratch (wound) assay analysis after 6 hrs. Representative images are shown, scale = 500 µm. (*n* = 3 independent biological replicates, 3 technical of each). (**D**) Invasion assay were performed using stable miR-expressing HUVECs pre-treated with mitomycin c and then allowed to migrate for 12 hrs. Representative images are shown, scale = 200 µm (*n* = 3 independent biological replicates, 3 technical replicates of each). Throughout figure, all graphs are shown as mean ± SEM. ^*^ *p* < 0.05; ^**^ *p* < 0.01; by two-tailed Student’s *t* test.

We have previously established that miR-151a enhances motility of lung epithelial cells including lung cancer cells (specifically non-small cell lung cancer (NSCLC) [29]. Therefore, we evaluated if the demonstrated miR-151a effect in lung cancer cell lines could be replicated in the human umbilical vein endothelial cells (HUVECs), which are often used to assess endothelial cell function. We first verified that miR-151a is overexpressed (approximately 4 fold) in miR-151a HUVEC and that anti-miR-151a reduces miR-151a expression (40–50%), relative to miR control cell lines (Figure 2A). miR-151a-overexpressing HUVECs showed a significant decrease in TEER relative to control HUVECs; in contrast neutralization of endogenously expressed miR-151a (by anti-miR-151a) resulted in enhanced TEER, compared to miR control cells (Figure 2B).

We next investigated the potential role of miR-151a on angiogenic properties of endothelial cells. We analyzed the role of miR-151a on migration and invasion of miR-modulated HUVECs. In order to separate cell motility from cell growth, we pretreated cells with Mitomycin c for 30 min and then subjected cells to wound healing and trans-well migration assays. miR-151a-overexpressing HUVECs showed enhanced migration, as determined by both the wound healing and transwell migration assays, relative to controls (Figure 2C, 2D). In contrast, anti-miR-151a decreased HUVEC migration relative to cells expressing the miR control (Figure 2C, 2D). Therefore, miR-151a overexpression promotes migration and invasion in HUVEC cells similar to our previous demonstrated effect in NSCLC, while anti-miR-151a inhibits these effects and promotes acquisition of endothelial barrier properties by neutralizing the endogenous miR-151a.

### miR-151a enhances angiogenesis

**Figure 3:**
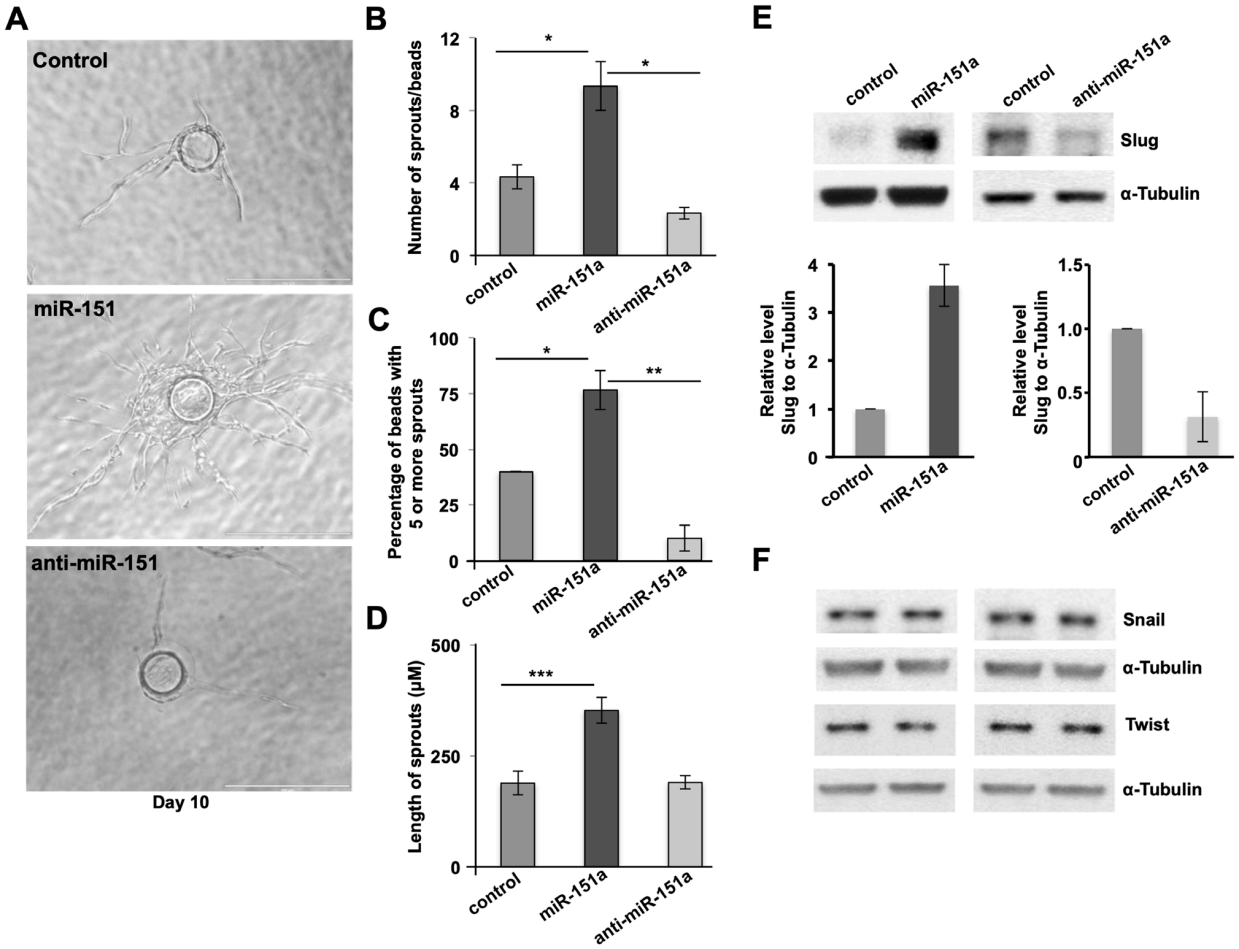
miR-151a enhances EC angiogenesis and induces the amount of Slug protein. (**A**) Stably miR-modulated HUVECs (miR control, miR-151a and anti-miR-151a) were subjected to *in vitro* angiogenesis bead assays. Nascent sprouts are observed on day 3–4 and cells continue to proliferate, migrate, branch and form lumens through day 6–10. Representative images depicting HUVEC cell angiogenesis in fibrin gels from day 10 are shown. Scale bars: 150 μm. Number of sprouts per bead (**B**) percentage of beads with 5 or more sprouts (**C**) and length of spouts (**D**) are shown (*n* = 3 independent biological replicates, 10 technical replicates of each) (**E**) Western blot analysis of Slug protein levels in miR-151a modulated HUVEC (top), and quantified relative to α-tubulin protein levels (bottom panels). (**F**) Western blot analysis of Snail and Twist protein levels in miR-151a modulated HUVEC were analyzed using α-tubulin as an internal control. Throughout figure, *n* = 3 independent biological replicates and graphs are shown as mean ± SEM. ^*^ *p* < 0.05; ^**^ *p* < 0.01; ^***^ *p* < 0.001; by two-tailed Student’s *t* test.

In order to determine whether miR-151a directly affects the formation of new blood vessels, we subjected miR modulated (miR control, miR-151a and anti-miR-151a overexpressing) HUVECs to the classical fibrin gel bead angiogenesis assay, in which endothelial angiogenesis can be analyzed for a period of 10 days in culture by measuring the number of endothelial cell sprout per bead and the length of newly-generated blood vessels (Figure 3A) [30]. Induced miR-151a expression significantly enhanced the endothelial cell angiogenesis potential by increasing both the number of sprouts per bead and the length of vessel sprouts, relative to miR control expressing HUVEC (Figure 3A–3D). In contrast anti-miR-151a had the opposite effect on new vessel formation, as compared to controls (Figure 3B–3D).

The angiogenic effect miR-151a overexpression in HUVECs, suggested that miR-151a regulates gene products involved in EC migration and angiogenesis. Although, we have previously shown that miR-151a depletes lung cancer cells of E-Cadherin, an adherens junction protein [29] miR-151a likely targets distinct gene products in endothelial cells since they do not express E-Cadherin. We initially investigated whether miR-151a could also target the adherens junction protein Cadherin-5 (VE-Cadherin), as the miR-151a seed binding site in E-Cadherin is present in VE-Cadherin. However, miR-151a does not modulate VE-Cadherin protein levels in a consistent manner in HUVEC (data not shown).

Based on the established key function of the transcription factors Snail, Slug and Twist and the endothelial to mesenchymal transition (EndoMT), we tested whether miR-151a regulates these transcription factors. We found a significant increase in Slug protein levels in miR-151a-overexpressing HUVECs, relative to miR controls (Figure 3E left panel, quantification shown in bottom panel), whereas anti-miR-151a decreased Slug protein levels, compared to controls (Figure 3E right panel, quantification is shown in the bottom panel). miR-151a was found to specifically regulate Slug protein levels, as neither Snail nor Twist protein amounts were affected by either miR-151a or anti-miR-151a overexpression in HUVECs (Figure 3F). Since, miR-151a overexpression *increases* the level of Slug protein, this suggests that Slug is indirectly affected by miR-151a, through an unknown upstream signaling partner. Future research is needed to determine additional aspects of these pathways in endothelial cells during angiogenesis.

### miR-151a-induced angiogenesis is dependent on Slug

**Figure 4:**
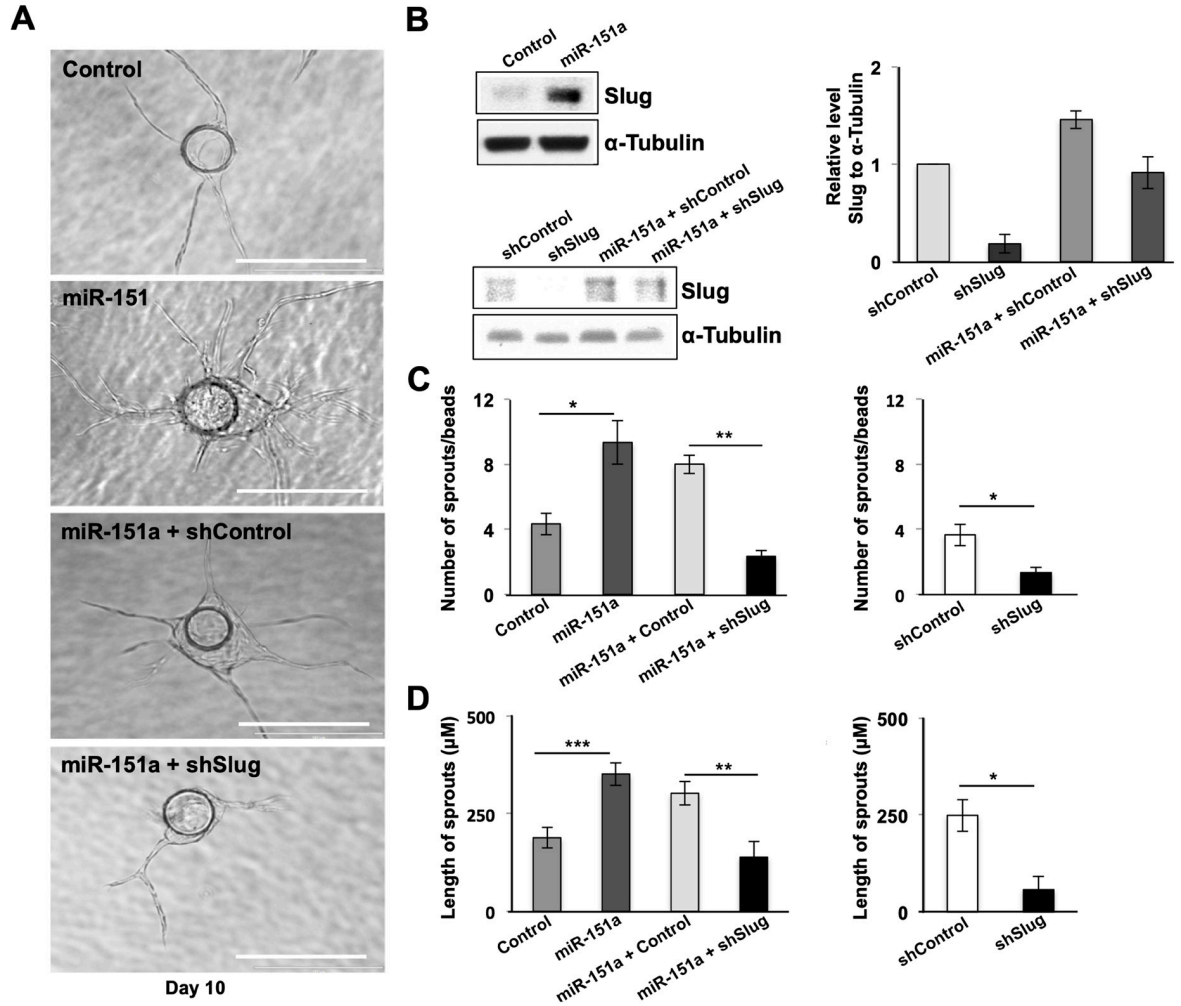
miR-151a enhanced EC angiogenesis is dependent on Slug induction. (**A** and **B**) Slug depletion was performed in control cells and in stably miR-modulated HUVECs (miR control, miR-151a) using sh-Slug relative to a sh-control. Effective Slug depletion was verified by Western blot analysis of Slug protein levels. Quantification of Slug knock-down relative to α-tubulin is shown (right panels). *n* = 3 independent biological replicates. miR-151a-overexpressing, miR control, sh-control, sh-Slug, miR-151a-sh-Slug and miR-151a-sh-Slug HUVEC were subjected to *in vitro* angiogenesis bead assays. Representative images depicting HUVEC cell angiogenesis in fibrin gels from day 10 are shown. Scale bars: 150 μm. Number of sprouts per bead (**C**) and (**D**) and length of spouts are shown (*n* = 3 independent biological replicates, 10 technical replicates of each). Graphs are shown as mean ± SEM. ^*^ *p* < 0.05; ^**^ *p* < 0.01; ^***^ *p* < 0.001; by two-tailed Student’s *t* test.

In order to address the relative importance of Slug in miR-151a induced angiogenesis, we next performed rescue experiment. We generated miR control and miR-151a HUVEC cell lines, in which we either expressed sh-control (sh-GFP) or (sh-Slug) lentivirus to specifically affect Slug levels in the context of miR-151a overexpression. We verified that Slug indeed was significantly induced by miR-151a as previously determined (Figure 4B and Figure 3E) and that sh-Slug significantly decreased Slug protein levels in sh-control and miR-151a overexpressing HUVEC cells (Figure 4B bottom panel, quantified in the right panel). We next performed bead angiogenesis assays and found that miR-151a-induced Slug expression is indeed required for miR-151a enhanced angiogenesis, since both the number of vessel sprouts from the EC-covered bead (Figure 4A and Figure 4C) and the vessel sprout length (Figure 4A and Figure 4D) were significantly reduced in miR-151a overexpressing HUVEC cells that harbored an sh-Slug lentivirus, but not the control sh-GFP control.

### miR-151a enhances angiogenesis in 3D vascularized tumor spheroids

**Figure 5:**
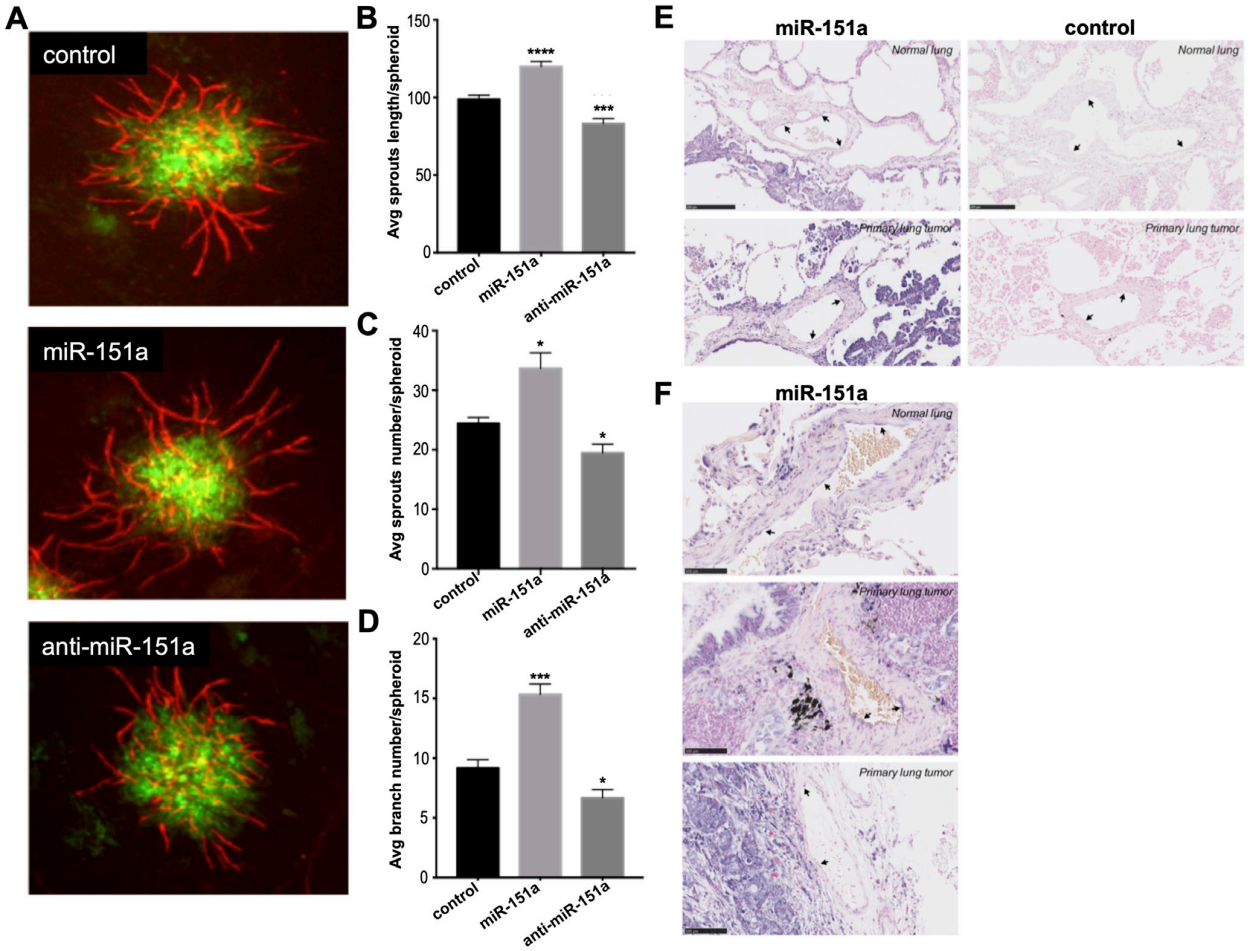
miR-151a enhances angiogenesis in 3D vascularized lung tumor spheroids and is expressed in vasculature of NSCLC patient specimens. (**A**) Stable miR-modulated (GFP-expressing miR control, miR-151a and anti-miR-151a) HUVEC and lung cancer cells (A549) were generated. miR-modulated lung cancer and endothelial cells were mixed and 3D vascularized tumor spheroids were allowed to form. On day 7 tissues were fixed and immunofluorescent staining was performed visualizing vessels (CD31, red) and tumor cells (GFP, green). Representative images from one of at least three similar experiments are shown. Scale bars: 150 μm. Average (Avg) sprout length per spheroid (**B**) average sprout number per spheroid (**C**) and average branch number per spheroid (**D**) are shown. (**E** and **F**) The expression level of miR-151a was analyzed by *in situ* hybridization in two normal lung samples and three primary NSCLCs (miR-151a expression: high = purple, low = light pink). Scale = 50 µm in Figure 5E and scale = 100 µm in Figure 5F. ****

In order to address whether miR-151a likely plays important roles both in lung endothelial cells and in lung tumors, we evaluated whether miR-151a affect angiogenesis in a 3D vascularized lung cancer spheroid assay. In brief, miR-GFP-expressing lung cancer cells (A549 cells-green) and HUVEC cells were mixed, vascularized spheroids were generated and fixed on day 7. Immunofluorescence staining was performed to visualize angiogenesis using an anti-CD31 antibody that labels endothelial cells (red) and tumor vessel networks were assessed, by counting the number of sprouts or by measuring the length of the vascular sprouts (Figure 5). We found that miR-151a modestly, though significantly, enhances angiogenesis in the lung cancer 3D spheroid model relative to miR controls, both in regards to average (avg) sprout length/spheroid (Figure 5B), average sprouts number/spheroid (Figure 5C) and average branch number/spheroid (Figure 5D). In contrast anti-miR-151a reduces angiogenesis, as compared to miR control spheroid (Figure 5A–5D). These finding suggests that miR-151a may play a possible role in lung cancer-associated angiogenesis.

### NSCLC patient specimens are characterized by low to medium endothelial cell miR-151a expression

In order to evaluate the physiological relevance of our *in vitro* 3D tumor-associated angiogenesis *in vitro* model, we next evaluated whether human lung blood vessels express miR-151a. We performed *in situ* hybridization of human miR-151a in human lung tissue and found that miR-151a is expressed at low to medium levels in both blood vessels of normal lung tissue, (Figure 5E top left, black arrows), as well as in primary tumor tissue from patients with non-small cell lung cancer (NSCLC) (Figure 5E and 5F, black arrows). In summary, our study supports the concept that miR-151a play important roles in the human lung, and that dysregulation of miR-151a levels may contribute to lung cancer pathogenesis.

## DISCUSSION

In the present study we demonstrate that endothelial cells both in the lung of either normal or lung cancer patients express miR-151a at similar levels. We find that induced miR-151a levels promote EC angiogenic properties such as increased motility and angiogenesis, and we determine that this process depends on increased Slug protein levels. We have previously reported that miR-151a functions as an onco-miR in NSCLC cells where miR-151a is highly over-expressed compared to normal lung epithelial cells [29]. A logical next question will be to address how miR-151a is regulated. In this context it is important to note that the lung cancer microenvironment is known to be inflamed, and it is possible that inflammatory cytokines are involved in the overexpression of miR-151a [31–37].

The mechanism by which miR-151a induces Slug protein levels needs further investigation. It is well established that Slug is a key transcription factor implicated in angiogenesis and the EndoMT transition by repressing E-Cad expression and inducing N-Cad expression, thereby effectively regulating the transition between EMT and MET [38, 39]. Slug has been reported to be overexpressed in many types of cancer, including lung cancer [40–46]. In addition, it is known that the family of EMT-TFs can be activated by a myriad of receptors and signaling pathways including tumor growth factor TFGβ, Notch, Wnt, morphogenetic protein BMPs, Ras/Erk pathways as well as inflammation, hypoxia and shear stress forces, and that the EMT-TF can also regulate each other in complex and interdependent ways [12, 47]. However, the actual molecular mechanisms that regulate Slug transcription are still unclear. Recently, an upstream activator of Slug was identified. This multifunctional protein, known as staphylococcal nuclease domain-containing protein-1 (SND1, or Tudor-SN, p100) activates Slug transcription through chromatic remodeling and has oncogenic properties including promotion of angiogenesis [48–51]. Based on the notion that miRs function by enhancing mRNA decay or by inhibiting protein translation, our results suggest that miR-151a increases Slug expression indirectly by modulating the expression of a putative negative regulator of Slug which still needs to be identified.

Interestingly, Slug has previously been reported to be overexpressed in blood vessels adjacent to invasive tumors, in contrast to the quiescent vasculature where Slug is generally absent [14]. Our finding that miR-151a is expressed at low to medium levels in blood vessels in lung cancer tissue from patients with NSCLC (Figure 5E), supports the idea that tumor-associated angiogenesis may be characterized by endothelial cell miR-151a expression, that results in Slug overexpression and potential EndoMT transition. We and others, have previously determined that miRs often function as master regulators by targeting multiple gene products in the same cellular pathway [23, 52–56]. Based on this hypothesis we predict that additional miR-151a targets will be identified and that these will add to our understanding of the mechanisms by which one miR can play key roles in complex cellular niches, such as the lung cancer niche.

Our results evaluating the effect of miR-151a expression in 3D vascularized lung tumor spheroids, supports the idea that miR-151a expression in the tumor environment is multibranched. Our previous findings have established a role for miR-151a as an onco-miR in lung cancer cells (NSCLC) resulting in enhanced tumor cell growth, motility and partial EMT [29]. Our current work indicates a role for miR-151a in tumor-associated endothelial cell angiogenesis. It will be important to begin to dissect the roles of miR-151a in tumor niche interactions. These studies may be able to address whether differential expression levels of miR-151a in tumor cells affects the heterogeneous nature of cancer cells in the tumor niche, including mitotic potential, hypoxic index, EMT/MET transition, potential to dedifferentiate to a stemness state etc. Finally, the role of heterogeneous miR-151a expression in different cell types within the niche and its effects on the interactions between tumor associated cells (including stromal cells, fibroblasts, inflammatory and immune infiltrating cells) and cancer cells will add needed understanding to the physiology of the tumor niche, which is required for the development of better targeted therapies.

In conclusion, our results show that increased miR-151 expression, significantly promotes endothelial cell motility and angiogenesis in 2D and 3D models, and that miR-151a-induced Slug expression is required for these endothelial cell properties. Our findings provide a new avenue to the understanding of the processes in the lung niche environment, and may facilitate the development of potential therapeutics against lung cancer.

## MATERIALS AND METHODS

### Patient samples

Formalin-fixed, paraffin embedded (FFPE) surgical specimens from 3 lung adenocarcinoma (NSCLC) patients, for more details [57]. The study was approved by the Regional Ethical Committee (Permission No.: 1-10-72-20-14) and all experiments were conducted in accordance with this approval.

### Cell culture and transductions

Cells were incubated at 37°C and 5% CO_2_ and routinely checked for mycoplasma contamination. Mouse lung endothelial cells (mLEC; C57-6011, Cell Biologics) were maintained in complete mEC media (M1168, Cell Biologics) and 10% FBS (FB-02, Omega Scientific). Human lung EC (hLEC; #3000, ScieneCell™) was maintained on plates coated with 10 µg/ml fibronectin (F2006, Sigma-Aldrich) in EC media (1001, ScienCell™). Primary human umbilical vein endothelial cells (HUVECs) were isolated from umbilical cords obtained from local hospitals under University of California Irvine Institutional Review Board approval. Alternatively, HUVECs were purchased (ATCC CRL-1730). HUVECs were routinely cultured in 1×M199 (Life Technologies) supplemented with 10% fetal bovine serum (FBS) and endothelial cell growth supplement (ECGS; BD Biosciences). Normal human lung fibroblasts (NHLF) were purchased from Lonza, routinely grown in 1×M199 supplemented with 10% FBS.

Plasmids used: siSlug3 (#10905, Addgene) plasmid was used for Slug depletion studies.

Stable transduction of miRs: VSV-G-pseudotyped lentiviral particles were made by transfecting 5.3 μg of pMD2-G (12259, Addgene), 9.7 μg of pCMV-DR8.74 (8455, Addgene) and 15 μg pCD510B-1 (miR Control), mZIP, pCD510B-1-miR-151a or mZIP-anti-miR-151a into 293T cells using Lipofectamine LTX (15338030, ThermoFisher). Viral supernatants were concentrated using PEG-it (LV810A-1, System Biosciences). Cells were transduced with high titer virus using polybrene (sc-134220, Santa Cruz Biotech) and spinoculated at 800 × g at 32°C for 30 minutes. Transduced cells were selected and maintained using 10 μg/ml puromycin.

### anti-miR library screen

The Electrical Cell-Substrate Impedance Sensing system (ECIS ZΘ; Applied BioPhysics) was used to monitor transendothelial electrical resistance (TEER) in real time in 96-well plate format (96W20idf PET plates, Applied Biophysics). The plates were stabilized with 10 mM L-Cysteine (C6852, Sigma-Aldrich) coated with 100 μg/mL Poly-D-lysine (P6407, Sigma-Aldrich), then coated with 10 μg/mL fibronectin (F2006, Sigma-Aldrich). For all ECIS experiments, LECs were plated at 50,000 cells/well. TEER was measured at 4000 Hz for 72–96 hr. mLECs were plated at 10,000 cells per well in a 96-well plate, incubated with 8 μg/mL polybrene (SC-134220, Santa Cruz Biotechnology) and transduced by spinoculation (800 x g at 32°C for 30 min) with the miRZipTM anti-miR lentiviral library (MZIPPLVA-1, System Biosciences) using 10,000 IFUs per well to ensure an MOI<1. Puromycin selection (1 μg/mL) was initiated 48 hours after transduction with full media changes every second day. hLECs were transduced with the miR overexpression and knockdown high titer viruses and selected with 10 μg/ml puromycin after which an ECIS experiment was performed.

### *In situ* hybridization and immunohistochemistry

For *in situ* hybridization analysis of miR-151a expression, a 5′- and 3′-double digoxigenin-labeled miRCURY LNA™ microRNA Detection Probe (612499-360, Exiqon) was used following manufacturer’s protocol. Scrambled probe and U6 were included as controls. Briefly, the deparaffinized, proteinase K-digested and dehydrated sections were hybridized with 40 nM double-DIG LNA™ hsa-miR-151a-5p probe for 60 min at 50°C. After a series of stringent washes with saline-sodium citrate buffer, sections were blocked then incubated with 1:125 dilution of anti-DIG Fab fragments conjugated to alkaline phosphatase (11093274910, Roche Diagnostics). After 30 min, the anti-DIG/AP was repNSCLCed with fresh reagent and incubated for an additional 30 min. The signal was detected using freshly prepared NBT/BCIP AP substrate (11-681-451-001, Roche Diagnostics). After 60 min at 30°C, fresh AP substrate was added and incubated for 60 min. Finally, slides were counterstained with Nuclear Fast Red™ (H-3403, Vector laboratories). Immuhistochemical staining was performed routinely using the VENTANA BenchMark XT staining system (Roche Diagnostics) with antibodies against E-cadherin (790-4497, Ventana, Roche Diagnostics) and cytokeratin 7 (790-4462, Ventana, Roche Diagnostics) according to manufacturer’s protocol.

### *In vitro* migration and invasion assays

Cells were pre-treated with 10 µg/ml mitomycin c (BP25312, Fisher Scientific) for 2 hours. Confluent cells were scratched and imaged after 6 or 14 hours and the percent healed was calculated. Transwell migration assay: 6.5 mm transwells with (8 µm) inserts (3464, Corning) were coated for 2 hours with 10 µg/ml fibronectin (F2006, ThermoFisher). Migrated cells were fixed, stained with DAPI and counted. Invasion assay: 8 µm PET inserts coated with Matrigel (354480, Corning) were used. After 12 hours invasion was determined as in transwell experiments.

### *In vitro* fibrin gel angiogenesis (bead) assay

Fibrin gel angiogenesis assays were performed as described [14, 58]. Briefly, HUVECs (passage 3–4) were mixed with Cytodex 3 microcarrier beads (Amersham) at a concentration of 150 cells/bead for 4 hours at 37°C and are allowed to adhere overnight. HUVEC-coated beads were then resuspended in a 2.5 mg/ml fibrinogen solution (MP Biomedicals) at a concentration of 250 beads/ml. Gels were formed by adding 500 µl of the fibrinogen/bead suspension to each well of a 24-well plate containing 0.5 U of thrombin (Sigma-Aldrich). Once gels clotted, 1 ml of EMG2 containing 20,000–50,000 NHLF (lung fibroblasts) was added to each well. Assays were quantified between days 5 and 6 by live-culture imaging using bright-field microscopy. Ten beads per condition were quantified per experiment.

### 3D vascularized lung cancer spheroid assay

Tumor spheroid assays were performed as described [14]. In brief, HUVECs and A549 cells were seeded into EGM-2 containing 15% methylcellulose at 7.5 × 10^4^ cells/ml and 2.5 × 10^4^ cells/ml, respectively, plated in 96-well U-bottom plate (Greiner Bio-one, CellStar) and spheroid formation were allowed overnight. Spheroids were then resuspended in fibrinogen (2.5 mg/ml; Sigma) containing NHLF at 1 × 10^6^ cells/ml. 50 µl of spheroid/cell suspension was added onto a 12 mm circular glass coverslip with an affixed polydimethylsiloxane (PDMS)-retaining ring and mixed with 5 × 10^−3^ U thrombin (Sigma-Aldrich). Tissues were fed with EGM2 and maintained at 37°C and 5% CO_2_. On day 7, tissues were fixed and immunofluorescent staining was performed. Tumor vessel networks were assessed by counting the number of sprouts or by measuring the length of the generated sprout.

### Immunofluorescence

3D vascularized lung cancer spheroids were fixed in 10% formalin (Fisher Scientific). Tissues were permeabilized for 30 minutes at room temperature using 1×PBS supplemented with 0.5% Tween-20. Blocking buffer (1×PBS containing 2% BSA and 0.1% Tween-20) was then used to block non-specific binding. Tissues were incubated overnight at 4°C using a mouse anti-CD31 antibody (1:100; Dako, IR610) diluted in blocking buffer followed by a goat anti-mouse 568-conjugated (1:500; Invitrogen, A11004) secondary antibody also diluted in blocking buffer. Tissues were extensively washed with 1×PBS containing 0.3 M glycine to remove background. All steps were completed under gentle agitation.

### Western blot

Cells were lysed in RIPA buffer (89901, ThermoFisher) with inhibitor cocktail (PI78410, ThermoFisher). 4x LDS sample buffer (NP0008, ThermoFisher) was used, samples boiled at 95°C for 10 minutes. NuPAGE Novex 4–12% Bis-Tris Protein Gels (NP0335, ThermoFisher Scientific), PVDF membranes, Blocking (PBST 5% nonfat milk), primary antibodies anti-Slug (C19G7, #9585, Cell Signaling Technology,), anti-Snail (C15D3, #3879, Cell Signaling Technology), anti-Twist (ab50887, abcam), VE-Cadherin (Cell Signaling Technology, D87F2 #2500) at 1:1000 dilution or tubulin (ab4074, abcam) at 1:2000 dilution, secondary antibody (HRP-linked anti-rabbit IgG antibody, 7074S, Cell Signaling Technology) at 1:5000 dilution and Pierce ECL Western Blotting Substrate (32106, ThermoFisher) and Bio-Rad ChemiDoc XRS+ System were used for protein expression development.

### RNA isolation and RT-qPCR

For all cell lines, RNA extraction and RT-qPCR experiments were conducted as previously described [29]. miR expression analysis was performed using the miRCURY LNA Universal RT microRNA PCR system (203301, Exiqon, Woburn, MA, USA), according to manufacturer’s protocol. All RT-qPCR was performed in technical triplicates using RNU5A as a reference gene.

### Statistical analysis

Researchers were blinded to experimental conditions prior to performing quantifications. All experiments were repeated at least three times. Student’s *t*-tests were used to calculate two-tailed *p* values and data are displayed as mean ± standard error of the mean (SEM) of independent biological replicates, (n) as indicated.

## Abbreviations

ECIS: electric cell-substrate impedance sensing
ECM: extracellular matrix
EMT: epithelial-to-mesenchymal-transition
E-Cad: E-Cadherin
EC: endothelial cell
EndoMT: endothelial to mesenchymal transition
GFP: green fluorescent protein
HUVEC: human umbilical vein cell
miR: microRNA
mRNA: messenger RNA
MET: mesenchymal-to-epithelial-transition
mLEC: mouse lung endothelial cell
N-Cad: N-Cadherin
NSCLC: non-small cell lung cancer
shRNA: short-hairpin RNA
SND1: Staphylococcal Nuclease Domain-Containing Protein 1
TEER: trans-endothelial electrical resistance
